# Appeal and Accountancy Methods Examined

**Published:** 1916-01-22

**Authors:** 


### WAR-TIME CHARITIES.

### Appeal and Accountancy Methods Examined.

A purist in matters of institutional and charity accountancy is naturally a little surprised, when sending his modest contribution in answer to one or other of the many attractive war appeals now before the public, to receive in return no form of acknowledgment which meets with his ideal of what is correct in the circumstances; nor is he given, in most cases, an opportunity of inspecting any statement of accounts in reference to the charity or organisation he is helping to maintain.

If our purist happens also to be a worker in a metropolitan hospital another matter that gives him material for thought and perhaps envy is the seemingly almost unlimited sums that the war charities are able to spend upon appeals and advertisements. At his own home he receives quite frequently packets of appeal literature, the envelopes having evidently been addressed in thousands from directories, and he notes that in many cases the supplicants expend twice the usual cost of postage by enclosing a stamped envelope for reply. These appeals are costly, whether they are unduly so is another matter, and some older-fashioned charities, especially metropolitan hospitals, are placed at a disadvantage because they cannot, except in a small measure, use the same methods of appeal. This is so because the accounts of permanent institutions are subject to close criticism.

### Wide Publicity Essential.

A war charity, it may be claimed, however, is a temporary affair, one that will be wound up no doubt when the happy time of having no war arrives. Its success depends upon wide publicity, it works at such feverish haste while it is alive that methods of accountancy which might be proper with permanent institutions may be modified to meet the extraordinary situation. Into how far these claims are just it should be the business of some duly constituted authority to inquire.

The Commissioner of Police has had something to say in the matter; he has estimated that so many millions have been given by a generous public to the various funds, he has warned the community against impostors, has limited street collections, and his department has shown some practical interest by prosecuting a few misguided people who have taken illegal advantage of the easy opportunities of the times. Such are the only visible signs that the authorities are alive to the possibility of fraud. We have no means of judging if any of the charities we meet face to face so many times in each day are or are not properly administered beyond judging them by standards which we have always taught should properly be set up in such matters.

It is a difficult and ungrateful task in this connection to criticise at all, but when the criticism is applied by one newspaper to another the work presents many obstacles. Most of these war charities are provided directly with the means of subsistence by our great newspapers, and all credit and praise must be accorded to them for work which has been the means of relieving much suffering and making easier the lot of the brave soldiers and the poorer part of the population of this country and her Allies. In addition many other charities, more or less useful and desirable, could not exist without the publicity afforded by the advertisement columns of the newspapers.

### A Proper Form of Acknowledgment.

We are all familiar with the methods of the newspaper fund. Sums received are acknowledged in the news columns and nothing, as a rule, is sent direct to a donor unless it be in a form which would not be approved by an auditor who knows and enforces forms of accountancy of a particular standard as applied to charities. Why a proper form of receipt, taken from a book with a correct recording counterfoil or duplicate, cannot be sent to the individual donor, we have never been able to surmise. Such a proceeding would, one judges, be the only means of providing a reasonably satisfactory form of evidence to an auditor that the money has been received and accounted for.

Fortunately, in these cases we have a practical safeguard, and that is the honour and reputation of organs that are well known to and respected by the public. There may be other organisations who are more willing to issue very correct acknowledgments than to produce anything in the shape of an understandable account or balance sheet. These can get their revenue easily nowadays, anyone can obtain money by the use of the words "soldier," "ambulance," "wounded," etc., but how that money is spent is too often hidden behind a veil.

We look at a back issue of a great London daily and find in the "agony" column for one day fourteen public and seven private appeals. People are confidently asked to provide all kinds of garments to the soldiers of different regiments, to help to send necessaries to prisoners of war in foreign countries, to buy tobacco and cigarettes for the use of the soldiers on active service, to subscribe to the cost of entertainments to the wounded, to purchase material for the manufacture of sandbags and otherwise to augment the strain upon their private charity calls. Fortunately, to these advertisements are generally appended names of people whose position is a guarantee of good faith, so far as that may be a satisfaction to the person who uses prudence in his almsgiving.

We have never been able to understand why the law directs the prosecution of a person who asks for alms in the street and at the same time allows another kind of beggar to do the same thing through the "agony" column of a newspaper. We find in the print we refer to on one day seven instances of the latter form of solicitation. The following is a specimen: "Officer leaving for the Front is anxious to meet someone who will guarantee his mother and sister the necessaries of life while away." The person who inserts this advertisement may or may not be an officer, and leaving for the Front. If he really is an officer, he ought to know that it is not consistent with his position to do this sort of thing. In any case, he will observe a few inches above his own announcement one of a charity requesting contributions towards an Officers' Family Fund, and there should have been some channel by which he could place his unfortunate position before a body which has seemingly been inaugurated to meet the needs of just such cases as his. That brings us to consideration of the fact that there is no co-operation and co-ordinance between these temporary charities. Many of them have precisely the same objects, and the waste of administrative expense through overlapping must be very considerable.

The expenditure on administration, including, as it does, the cost of raising funds, is obviously very high. We notice in one issue of a daily newspaper two whole columns devoted to six large displayed advertisements of war charities; and in other issues many columns devoted to acknowledgments on behalf of the Serbian Red Cross, the Polish Victims' Relief Fund, the Russian Prisoners of War Fund, the French Wounded Emergency Fund, and the Italian Wounded Fund; and these represent but a small fraction of the space devoted to advertisements of this category.

The taste of some of the announcements is a little peculiar. Who would consider that the following verse (one of three taking up quite a lot of space) would be worthy of reproducing almost daily for some weeks?

I'm only a cavalry charger,
And my eyes are becoming quite dim
(I really don't mind though I'm "done for"
So long as I'm going to *him*) :
But first I would plead for my comrades,
Who're dying and suffering too!
Oh, please help the poor wounded horses :
I'm sure that you would if you knew.

There is this to be said for our newspapers: that the verses would never have a ghost of a chance of appearing in any but the advertisement pages. To sum up, we venture to advance the suggestion that there is room for an official censor of war-fund advertisements and an official inspector of war-fund accounts, and that both of these are quite necessary to the interests of a generous but not always discriminating public.

